# Programmed Topographic Substrates for Studying Roughness Gradient-Dependent Cell Migration Using Two-Photon Polymerization

**DOI:** 10.3389/fcell.2022.825791

**Published:** 2022-03-22

**Authors:** Subhashree Shivani, Yu-Hsiang Hsu, Cheng-Je Lee, Chi-Sheng Cheong, Tien-Tung Chung, An-Bang Wang

**Affiliations:** ^1^ Institute of Applied Mechanics, National Taiwan University, Taipei, Taiwan; ^2^ Department of Mechanical Engineering, National Taiwan University, Taipei, Taiwan

**Keywords:** cell migration, topotaxis, contact guidance, two-photon polymerization, alignotaxis

## Abstract

The mediation of the extracellular matrix is one of the major environmental cues to direct cell migration, such as stiffness-dependent durotaxis and adhesiveness-dependent haptotaxis. In this study, we explore another possible contact guidance: roughness dependent topotaxis. Different from previously reported studies on topotaxis that use standard photolithography to create micron or submicron structures that have identical height and different spatial densities, we develop a new method to programmatically fabricate substrates with different patterns of surface roughness using two-photon polymerization. Surface roughness ranging from 0.29 to 1.11 μm can be created by controlling the voxel distance between adjacently cured ellipsoid voxels. Patterned Ormocomp^®^ masters are transferred to polypropylene films using the nanoimprinting method for cell migration study. Our experimental results suggest that MG63 cells can sense the spatial distribution of their underlying extracellar roughness and modulate their migration velocity and direction. Three characteristic behaviors were identified. First, cells have a higher migration velocity on substrates with higher roughness. Second, cells preferred to migrate from regions of higher roughness to lower roughness, and their migration velocity also decreased with descending roughness. Third, the migration velocity remained unchanged on the lower roughness range on a graded substrate with a steeper roughness. The last cell migration characteristic suggests the steepness of the roughness gradient can be another environmental cue in addition to surface roughness. Finally, the combination of two-photon polymerization and nanoimprint methods could become a new fabrication methodology to create better 3D intricate structures for exploring topotactic cell migrations.

## Introduction

Cells change their morphology and motility in response to biophysical and biochemical cues of the extracellular matrix (ECM) through a phenomenon called contact guidance (Richert et al., 2008; McMurray et al., 2011; Gattazzo et al., 2014; Zhao et al., 2018). Different substrate features such as topography (Kim et al., 2009a; Jeon et al., 2010) and stiffness (Engler et al., 2006; Wang et al., 2012) can control and influence cellular functions such as morphology, orientation, migration, proliferation, and differentiation. Unidirectional cell migration is vital for various physiological activities and can be regulated by the graded adhesion of the underlying matrix (haptotaxis) (Carter, 1965; Motta et al., 2019) or stiffness of the substrate (durotaxis) (Lo et al., 2000; Nasrollahi et al., 2017). For example, fibroblasts preferred to migrate towards higher ECM densities and stiffer areas of substrates. As the *in vivo* surfaces are more complex with multiple three-dimensional ECM features of different scales and sizes, current understanding of cell–ECM interactions is still limited. To better imitate the *in vivo* environment and understand the effect of topographic cues provided by the substrates, quasi-3D micro-patterned substrates created on 2D surfaces are recently used to study cell behaviors. Many eukaryotic cells have shown the potential to recognize these nano/micro-features by biasing their orientation and migration. Reported methods include 2 μm cross-ribs with several micron-scale intervals (Kim et al., 2009b), graded 1–3 μm micropost array with micron-scale spacing (Sochol et al., 2011), graded 600 nm nanopost array with 0.3–4.2 μm spacing (Park et al., 2016), and graded 280 nm nanoholes and nanopillars with submicron pitch (Cheng et al., 2021). This type of contact guidance is suggested as the new taxis phenomenon termed as topotaxis (Kim et al., 2012). Park et al. (2018) summarized the recent findings on topotaxis.

The above mentioned studies have successfully directed cell migration using topographic features. However, there are two complications induced by the design of these topographic features. First, the materials used were polydimethylsiloxane (PDMS) and UV-curable polyurethane acrylate (PUA). They are relatively soft materials with stiffness ranging between 0.5 MPa to several MPa. Thus, microposts and nanopillars can bend or deform due to traction force applied by a migrating cell (Viela et al., 2016; Liang et al., 2017). Thus, these micro- or nanostructures can provide different levels of equivalent stiffnesses to their attached cells. This effect has been demonstrated by Sochol et al. (2011) where bovine aortic endothelial cells tend to migrate from microposts with lower stiffness to higher stiffness. Second, these studies fixed the topographic feature and only vary the pitch distance between topographic features. This design results in the flat-to-feature ratio varied from 2 to 7 times, which means cells were not always in contact with the 3D micro- or nano-features.

To reduce possible complications induced from deformable micro- or nano-features and the intermediary flat area, we use the two-photon photolithography (TPP) method (Schizas and Karalekas, 2011) to create a topographic substrate on a biocompatible Ormocomp^®^ resin and transferred the design to a polypropylene (PP) film. Since the stiffness of PP is 1.32 GPa and the shape of micro-features are ellipsoid, the micro-features cannot bend or deform by cell tractions and a much uniform three-dimensional (3D) topographic structures without flat surfaces can be created. Thus, a topographic substrate that can simulate 3D topographic structures for topotaxis is created for this study.

TPP is a rapid photolithography method that can provide a localized polymerization in time and space to fabricate different 2D and 3D structures with a focused laser beam (Maruo et al., 1997; Kawata et al., 2001; Malinauskas et al., 2010). The spatial selectivity of two-photon absorption can be used to fabricate 3D polymeric structures with micron resolution. Particularly, TPP creates ellipsoid-shaped voxels at the focus point of laser beam and no sharp edges are introduced in the geometry of cured resin (Zhou et al., 2015). Thus, TPP can provide a more 3D biomimetic structure by controlling voxel distance between each laser pulse. Multiple studies have harnessed this property of TPP to fabricate scaffolds with microstructures for biomedical applications (Ovsianikov et al., 2007; Schlie et al., 2007; Claeyssens et al., 2009).

In this study, we present surface roughness R_z_ as a topotactic cue to bias migration of MG63 cells on a highly biomimetic ECM substrate using TPP. The developed substrates were precisely fabricated to avoid sharp edges and flat surfaces, and the density of R_z_ varied isotropically in both *x*-axis and *y*-axis. For gradient substrates, the R_z_ decreased continuously in the *x*-axis without any intermediate flat area. MG63 is an osteosarcoma cell line that has been widely used for understanding the interaction of bone cells and substrates, such as adhesion, migration, and proliferation on titanium (Deng et al., 2014; Vandrovcova et al., 2014; Liu et al., 2017). To replicate the mechanical properties of the *in vivo* bone tissues better and also avoid the deformation of topographic features, we developed a nanoimprinting process to transfer TPP fabricated Ormocomp^®^ molds to a polypropylene (PP) film. Using this method, four PP substrates with different roughness designs were fabricated. The uniformly roughened substrates were designed to analyze the effect of high and low roughness on MG63 cell migrations where cells showed higher migration speed on higher roughness. The other two substrates with roughness gradient were designed to study its effect on promoting unidirectional cell migrations. The developed TPP fabrication method for creating roughness gradient substrates can be found in our previously reported study (Chung et al., 2018). Here, we focus on the investigation of the contact guidance of MG63 cells on substrates with different R_z_ patterns.

## Materials and Methods

### Fabrication of Ormocomp^®^ Masters Using Programmed Voxel Distances

**FIGURE 1:**
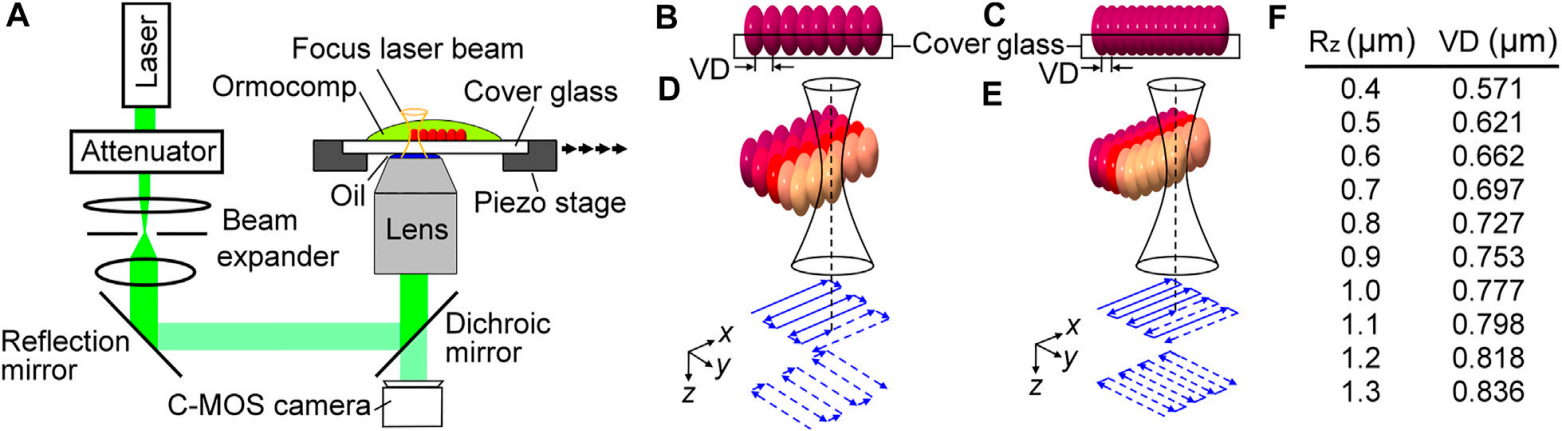
Illustrations of the TPP setup **(A)**, substrates with high surface roughness using a large voxel distance **(B)** and low surface roughness using a small voxel distance **(C)**, route patterns of raster programs designed for high **(D)** and low surface roughness, and a list of quantified correlations between R_z_ and low surface roughness **(E)**, and a list of quantified correlations between R_z_ and voxel distances **(F)**.

Fabrication protocols and programs of TPP were developed and optimized to create different R_z_ on Ormocomp^®^. To fabricate master molds, we changed the voxel distance between each cured voxel under a focused laser beam to create different roughness patterns as shown in Figure 1A. First, a low-cost double frequency Nd:YAG 532 nm green light laser was used to generate a 26 mW and 130 kHz pulse laser source. The power of the laser pulse was controlled by an attenuator, and a beam expander was used to expand and collimate the laser beam followed by coupling to an inverted microscope (OLYMPUS IX51) using a reflection mirror and a dichroic mirror. A 50x objective lens (Olympus MPLFLN50X) and standard lens oil was used to focus the laser beam near the top surface of a 170 nm cover glass that was covered with Ormocomp^®^ resin. The cover glass was placed on a high precision *x*-*y* motorized stage (PI, P-5453.C7) that was synchronized with the pulsating frequency of the laser. A C-MOS camera (EO-0413) was placed under the dichroic mirror to monitor the fabrication process in real-time. The motor moved with a programmed step size that was equal to the optimized voxel distance (VD), and each position was only exposed with a single pulse of the pulse laser. Using this method, each position had an ellipsoid-shaped cured voxel (red), and voxels were partially fused with adjacent voxels to create roughened topographic features. This concept can be understood from the side view of the substrates with large and small voxel distances as shown in Figures 1B,C, respectively. The level of partially fused voxels resulted in Ormocomp^®^ substrates with different surface roughness.

Applying this concept to TPP fabrication, we designed a raster program for the piezoelectric motor to create two masters with uniformly distributed low (Figure 1D) and high (Figure 1E) roughness. The piezoelectric motor first moved stepwise in the *x*-direction (top solid blue lines), and then moved in the *y*-direction (bottom blue dashed lines), with a step size equal to the voxel distance required for the desired roughness. The roughness (R_z_) of the substrates is controlled by varying the overlap between consecutive voxels known as the voxel distance. The correlation between VD and R_z_ are summarized in Figure 1F. It was found that the range of R_z_ can vary from 0.4 to 1.3 μm by controlling the voxel distances from 0.571 to 0.836 µm. To investigate the migratory behavior of MG63 cells, we set the overall TPP processing area to be 400 µm long by 200 µm wide. The *x*-axis and *y*-axis are defined in parallel with the 400 µm side and 200 µm side, respectively.

### Designing Topotactic Masters

To investigate the effect of surface roughness on cell motility, two substrates with uniform roughness were designed and fabricated, one with low R_z_ (U_LR_) and the other with high R_z_ (U_HR_). The optimized VD of U_LR_ and U_HR_ was 0.571 and 0.836 µm, respectively. It was found that their surface roughness R_a_ did not have significant difference. In contrast, the surface roughness R_z_ could adequately represent the roughness difference created by varying VD. The actual R_z_ of each substrate was quantified by analyzing confocal images. R_z_ is the average distance from the peak to valley of five highest peaks and five lowest valleys. As R_z_ can be influenced by extremes, areas with defects were avoided, and at least ten regions of 20 μm length in the *x*-direction (R_z_)_x_ and *y*-direction (R_z_)_y_ were sub-sampled and measured. Minor difference in R_z_ between the mold and substrate was observed, which could be attributed to the molding process.

The resultant R_z_ of the U_LR_ substrate was (R_z_)_x_ = 0.29 μm and (R_z_)_y_ = 0.49μm, and the resultant R_z_ of the U_HR_ substrate was (R_z_)_x_ = 1.1 μm and (R_z_)_y_ = 1.11 μm. To further study the influence of surface roughness on the unidirectional migration of MG63 cells, we designed two roughness gradient substrates. The first substrate was named G_40_ as it had 40 µm step size and 10 stairs along *x*-direction. Its R_z_ was varied from (R_z_)_x_ = 0.39–0.91 µm and from (R_z_)_y_ = 0.37–0.84 µm along with the stairs. The other substrate was named G_20_ as it had 20 µm step size. Its R_z_ was varied from (R_z_)_x_ = 0.30–0.78 µm and from (R_z_)_y_ = 0.31–0.99 µm along with the stairs. Two stair types were designed for G_20_ along *x*-axis: One with five stairs and the other with nine stairs.

### Fabrication of Topographic Substrates

Once these four Ormocomp^®^ masters were fabricated, and the designed topographic patterns were transferred to 75 µm-thick biocompatible polypropylene (PP) films using a two-step nanoimprint process. Young’s modulus of PP is 1.32 GPa, and it is close to the human proximal tibia (1.30 GPa) (Williams and Lewis, 1982). Thus, the influence of substrate stiffness was minimized and a better bone-like ECM was created. The developed two-step nanoimprint process is summarized as follows: First, Ormocomp^®^ masters were nanoimprinted onto a 75 μm thick PP film at 165°C and 50 psi for 20 min. These molded PP films served as the secondary masters. Then, an aluminium washer was fixed around the PP master followed by transferring to a polydimethylsiloxane (PDMS) sheet using PDMS micro-molding process. After curing at 55°C for 4 h, the washer with the enclosed PDMS mold was removed from the PP master. The PDMS mold served as the third master to transfer the patterns onto another 75 μm thick PP film by nanoimprint. The second PP film served as the final substrate for topotaxis study, and it had an inverse pattern of its Ormocomp^®^ master. The washer was used to prevent expansion of PDMS during nanoimprinting and ensured proper pattern transfer. The surface topography of nanoimprinted PP substrates was analzsed using a 3D laser scanning microscope (KEYENCE, VKX-210). It used the technology of confocal optics to provide a high vertical resolution at 0.5 nm, and is sufficient for the quantification of surface roughness. After the surface roughness was quantified and confirmed, the second PP films were sputter coated with 30 nm SiO_2_ on top of a 30 nm Ti adhesion layer. Then, each PP film was attached in a Petri dish followed by placing a 2 mm thick PDMS ring with 3 mm inner diameter just around the nanoimprinted area to create a culture well. Uncured PDMS mixture was used as an adhesive and was cured in a 55°C oven. The SiO_2_ coating on the topotactic substrate served as a charged surface to enhance the efficiency of fibronectin coating for cell adhesions.

### Cell Culture and Seeding

MG63 cells were cultured in CO_**2**_ independent medium (Gibco) supplemented with 10% fetal bovine serum (Biological Industries), 1% sodium pyruvate (Gibco), 1% Penicillin /Streptomycin (Lonza) and 1% L-Glutamine (Gibco). Before experiment, the entire setup of each substrate was sterilized under a UVC light for 8 h. The PP substrates were coated with 0.025 mg/ml fibronectin (Calbiochem 341668) in phosphate-buffered saline (PBS) for 1 h before cell loading. Then, cells were trypsinized and counted followed by seeding on to the device. The loading density were 2,500 cells/cm^2^, which is equivalent to 1 cell per 200 µm by 200 µm square area. Loaded devices were placed in a 37°C /5% CO_2_ incubator for 2 h to allow cell attachment before continuous imaging.

### Imaging

For long-term imaging on an inverted microscope, a lab-made aluminium (Al) bioreactor with dimensions of 50 mm in diameter and 10 mm in height was created. The bioreactor was filled with sterile DI-water to maintain humidity and had a transparent PMMA window for optical access. A ring-type film-heater was attached at the bottom Al plate, and a thermal couple was placed inside the DI-water. A P-I-D controller and a DC power supply was used to control and maintain the temperature at 37°C by tuning the heater with temperature feedback. Cell loaded device was placed in the bioreactor, surrounded by the sterile DI-water. Then, the bioreactor was placed on an inverted microscope (Olympus IX71) to monitor cell migration on each designed substrate for 15–18 h with 10 min interval. To investigate the influence of surface topography on cell migrations, only solo migrating cells that did not migrate along substrate edges or contacted other cells were investigated. Furthermore, only migratory cells that did not go into cell division were considered. At least 3–5 independent experiments were conducted for each topotactic substrate, and 5–9 independent cells were monitored and analyzed. After experiments, cells were fixed with standard fixation protocols followed by fluorescent staining protocols. Cell nuclei and F-actin networks were stained with blue fluorescent Hoescht 33342 (ThermoFisher, H3570) and rhodamine phalloidin (Life science, R415), respectively. The focal adhesion complex was immune-stained with FITC labeled anti-vinculin antibody (Sigma-Aldrich F7053) and GFP anti-paxillin antibody (Invitrogen Tyr-118).

### Statistical Analysis

Images were analyzed using ImageJ software, and Student’s *t*-test was used for statistical analysis, where *p* < 0.05 was considered significantly different. The migratory behavior of the cells was calculated by tracking their nuclei from time-lapse images. The velocity was calculated between every two time points, and the averaged velocity was calculated by data points retrieved from all the cells monitored from each topotactic substrate. The orientation angle (θ) was measured between the *x*-axis and the direction of cell migration, which positive orientation angle refers to the migration of cells upward from the *x*-axis and the negative orientation angle is downwards from the *x*-axis. The change of orientation angle (dθ) was quantified between data points of measured θ values, and the number of incidents were analyzed using 10- degree and 4-degree intervals for uniform (U_LR_ and U_HR_) and graded substrates (G_40_ and G_20_), respectively.

## Results

### Quantification of Topotactic Substrates With Uniform Roughness

**FIGURE 2:**
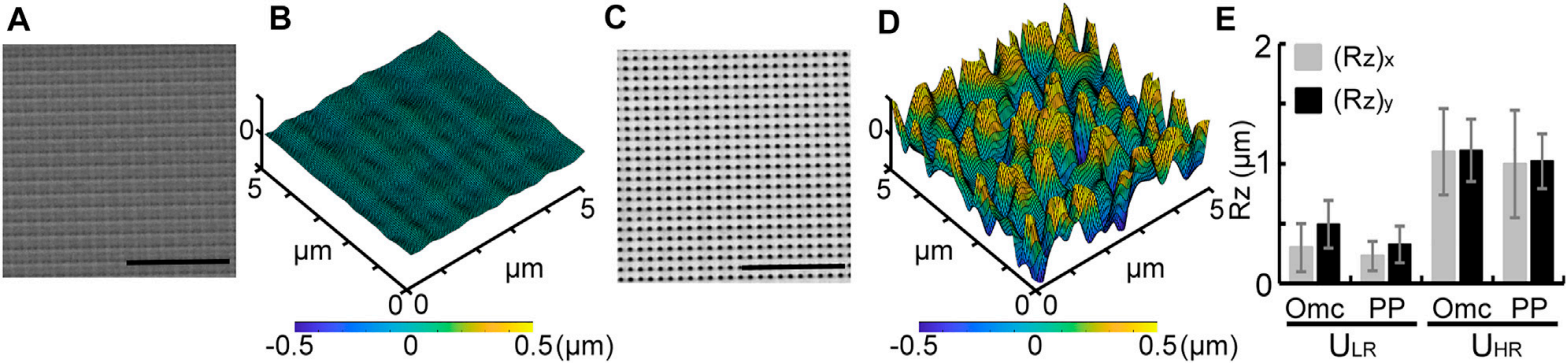
SEM micrographs of Ormocomp^®^ masters and 3D confocal scanned results of PP substrates for U_LR_ **(A–B)** and U_HR_ **(C–D)**. Scale bar = 10 μm **(A, C)**. Measured R_z_ values of Ormocomp^®^ masters and nanoimprinted PP substrates for U_LR_ and U_HR_ **(E)**.

Figures 2A–D show scanning electron microscope (SEM) micrographs of the Ormocomp^®^ masters and the confocal scanned results of U_LR_ and U_HR_ substrates, respectively. The surface topography with identical voxel distance produced uniformly roughened substrates. Their R_a_ values were not significantly different (Supplementary Figure S1A), and it was not sufficient to represent their topographic difference. In contrast, the surface roughness R_z_ showed a significant difference. Figure 2E shows the measured average (R_z_)_x_ and (R_z_)_y_ values for both Ormocomp^®^ masters (Omc) and the final PP substrates (PP). The measured actual R_z_ in both axes showed no significant difference, suggesting uniform roughness created by TPP and adequately replicated using nanoimprint. For Ormocomp^®^ masters, the quantified average R_z_ on U_LR_ was (R_z_)_x_ = 0.29 ± 0.2 μm and (R_z_)_y_ = 0.49 ± 0.2μm, and the R_z_ on U_HR_ was (R_z_)_x_ = 1.1 ± 0.36 μm and (R_z_)_y_ = 1.11 ± 0.26 μm. For PP substrates, the quantified average R_z_ for U_LR_ was (R_z_)_x_ = 0.23 ± 0.12 µm and (R_z_)_y_ = 0.33 ± 0.16 µm, and the R_z_ on U_HR_ was (R_z_)_x_ = 0.99 ± 0.45 µm and (R_z_)_y_ = 1.01 ± 0.22 µm. These results clearly demonstrate that uniformly roughened substrates with different R_z_ can be programmed and fabricated using the TPP technology.

### Migratory Behavior of MG63 Cells on Substrates With Uniform Roughness

**FIGURE 3:**
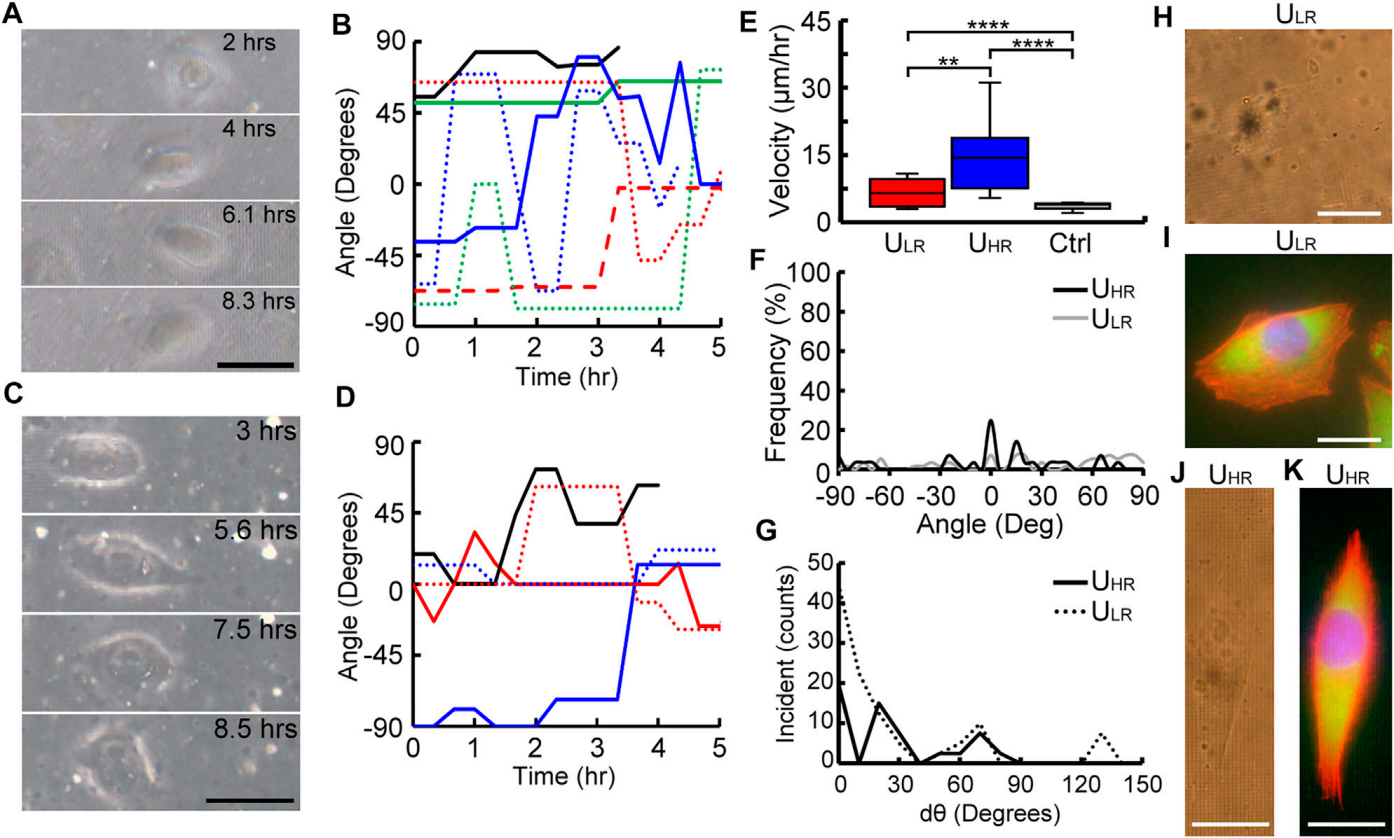
Analysis of cell migratory behaviors on uniform substrates. Time-lapse images and traces of orientation angle of cells migrated on ULR **(A–B)** and UHR **(C–D)** substrates. Comparison of range of migrating velocity **(E)**, orientation frequency **(F)**, the change of orientation angle dθ **(G)**, and phase contrast and fluorescent micrographs of cells migrated on ULR **(H–I)** and UHR **(J–K)** substrates, where nuclei, F-actin, and focal adhesion complex were stained with H33342 (Blue), rhodamine phalloidin (orange–red), anti-vinculin antibody, and anti-paxillin antibodies (green) Scale bar =100 µm.

Figure 3A shows the time-lapse images of a cell migrating on the U_LR_ substrate and represents the general motility behavior of cells migrating on this substrate. The cell did not show any preferred migration direction and had a relatively random orientation for the initial 8.3 h after seeding. The recording of the migration of this cell is shown in the Supplementary Video S1A. The contrast of these phase images was relatively low. It was because light scattering induced by the ellipsoid voxels that constructed the roughened substrate. Figure 3B shows the traced angle of orientations of 8 cells on the U_LR_ substrate 5 h after cell attachment. The *x*-axis was designated as 0° and the orientation of cells at each time points was traced with reference to this axis. We observed random migration of cells on U_LR_ substrates where the angle of orientation spanned between −90° and 90°.

For higher roughness, Figure 3C shows time-lapse images of a MG63 cell migrating on the U_HR_ substrate for the initial 8.5 h after seeding, and its recording can be found in the Supplementary Video S2C. Similar to U_LR_, the cell also migrated randomly on U_HR_. This behavior was confirmed by tracing the angle of orientation of five observed cells (Figure 3D). The migration velocity of the cells cultured on the two designed substrates and a flat substrate (Ctrl) were quantified and compared in Figure 3E. The cells showed significantly higher migration velocity on the U_HR_ (26.33 ± 15.17 μm/h) than on the U_LR_ (9.21 ± 5.98 μm/h) substrates, and they both were significantly faster than the cells on flat substrates (4.7 ± 1.65 μm/h).

The total range of angles covered by individual cells at each time point are plotted as occurrence frequency percentage in Figure 3F. As the cells migrated randomly with no preferred direction, expectedly, a wide range of orientation angle spanning between −90° and 90° was observed on both the substrates. We also quantified the change in orientation angle (dθ) in Figure 3G, where the range of dθ spanned from 0° to 140° and 0° to 90° for U_LR_ and U_HR_, respectively. This result clearly demonstrated the randomness of cell migration on these two uniform roughened substrates. Finally, phase images and fluorescent images of cells migrated on the U_LR_ and U_HR_ substrates are shown in Figures 3H–K, respectively. Their migration behaviors can be observed from their F-actin structures and distribution of focal adhesion complex. As observed, the cells on U_LR_ (Figure 3I) show a wide lamellipodium and a small tail suggesting that the migrating velocity is relatively low. Also, it has many lamellipodium protrusions on the leading edge suggesting that it has a less tendency to have a unidirectional migration. In contrast, the cell on U_HR_ (Figure 3K) has spindle morphology with an elongated lamellipodium with a couple of filopodia at the leading edge and a longer tail at the rear end. This is an indication of higher migration speed.

### Quantification of Topotactic Substrates With Graded Roughness

**FIGURE 4:**
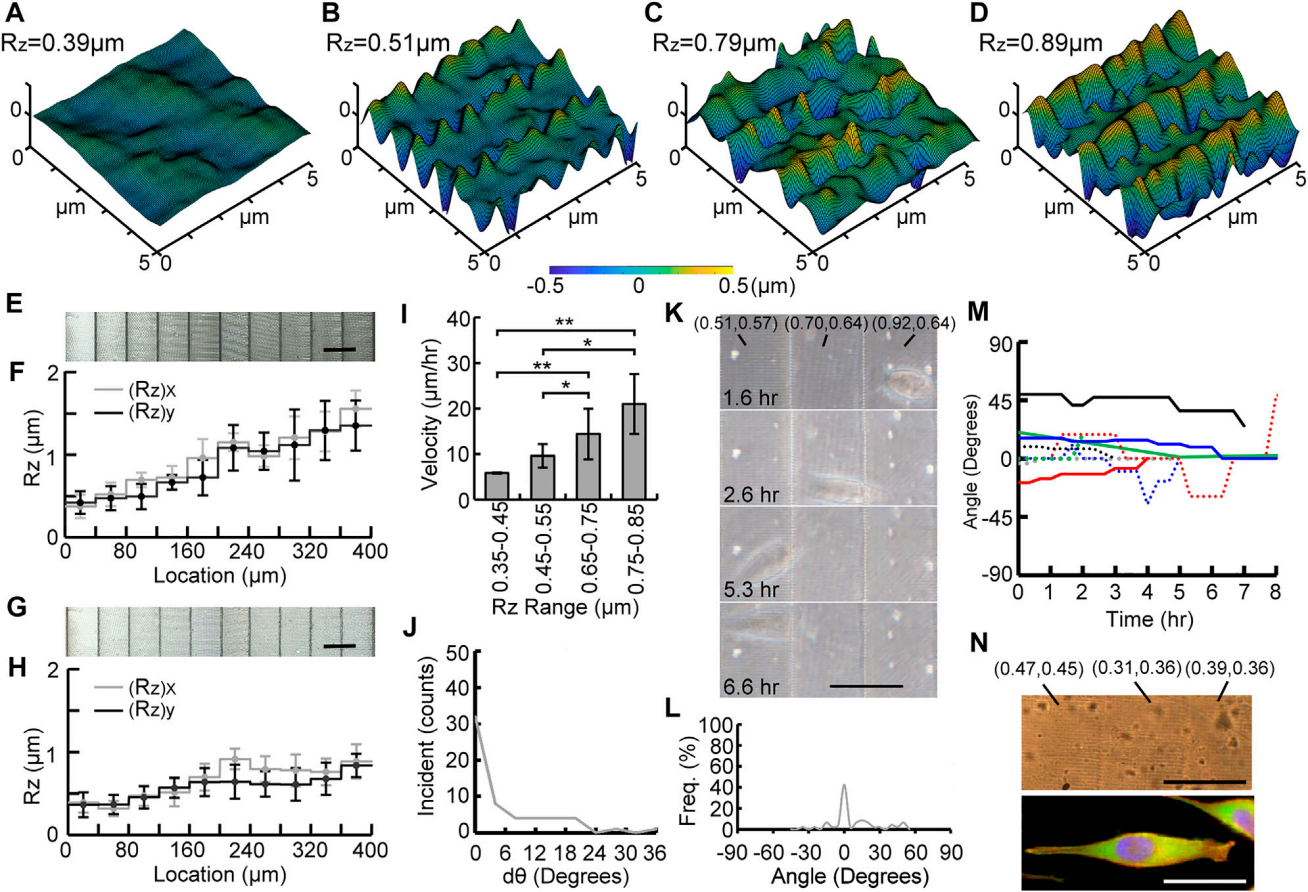
Experimental results of the G_40_ substrate. **(A–D)** confocal scanned profiles of four different roughness areas on G_40_. SEM micrographs and distributions of R_z_ values of Ormocomp^®^ substrates **(E–F)** and PP substrates **(G–H)**. Measured velocity on different roughness ranges of the substrate **(I)**, the change of orientation angle dθ **(J)**, time-lapse images of a migrated cell **(K)**, percentage of occurrence frequency of orientation angle **(L)**, traces of orientation angle of monitored cells (*n* = 8) **(M),** and phase contrast and fluorescent micrographs of a migrating cell on the G_40_ substrate **(N)**, where nuclei, F-actin, and focal adhesion complex were stained with H33342 (Blue), rhodamine phalloidin (orange–red), anti-vinculin antibody, and anti-paxillin antibodies (green) (scale bar = 40 μm).

**FIGURE 5:**
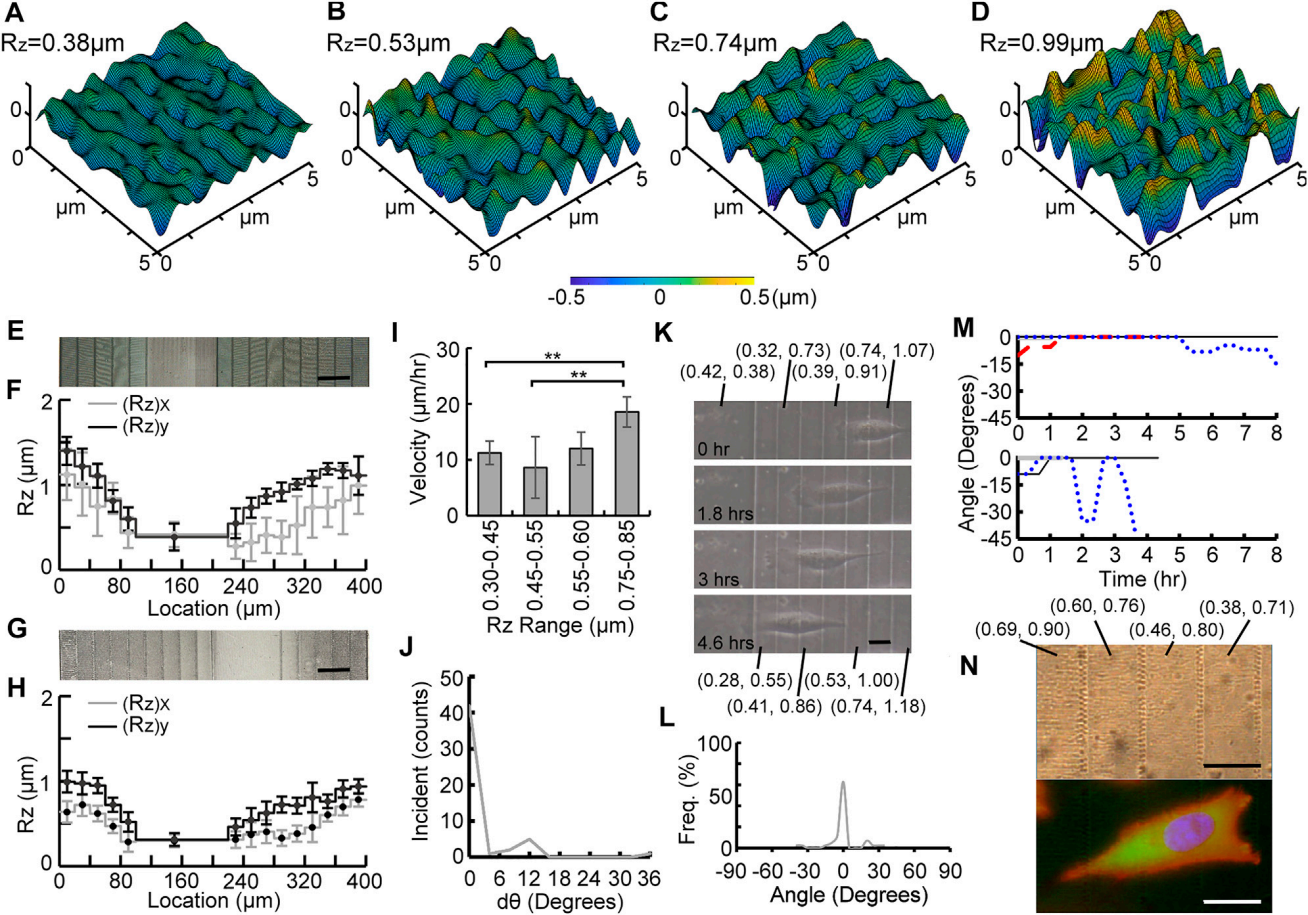
Experimental results of the G_20_ substrate. **(A–D)** confocal scanned profiles of four different roughness areas on G_20_. SEM micrographs and distributions of R_z_ values of Ormocomp^®^ substrates **(E–F)** and PP substrates **(G–H)**. Measured velocity on different roughness ranges of the substrate **(I)**, the change of orientation angle dθ **(J)**, time-lapse images of a migrated cell **(K)**, percentage of occurrence frequency of orientation angle **(L)**, traces of orientation angle of monitored cells (*n* = 7) **(M),** and phase contrast and fluorescent micrographs of a migrating cell on the G_20_ substrate **(N)**, where nuclei, F-actin, and focal adhesion complex were stained with H33342 (Blue), rhodamine phalloidin (orange–red), and anti-vinculin antibody and anti-paxillin antibodies (green) (scale bar = 40 μm).

Figures 4A–D, 5A–D show confocal scanned profiles of four different roughness areas on G_40_ and G_20_ gradient substrates, respectively. These results show that a gradient of continuously changing R_z_ without sharp edges can be created by varying voxel distance in the TPP method. Figures 4E, 5E show cropped SEM images of fabricated G_40_ and G_20_ Ormocomp^®^ masters, and measured R_z_ values are shown in Figures 4F, 5F, respectively. Similar to uniform substrates, measured R_a_ values between adjacent stairs of both graded substrates did not show any significant difference. (Supplementary Figures S1B, S1C). Figures 4G, 5G show the SEM micrographs of the corresponding nanoimprinted PP substrates, and their measured R_z_ values are shown in Figures 4H, 5H, respectively. The (R_z_)_x_ and (R_z_)_y_ values for G_40_ substrates ranged from 0.39 ± 0.12 µm to 0.91 ± 0.1 µm and from 0.37 ± 0.15 µm to 0.84 ± 0.13 µm, respectively. G_20_ substrates had two gradient designs on the 200 µm by 400 µm substrate: one with five stairs (left side in Figure 5G) and the other with nine stairs (right side in Figure 5G). The (R_z_)_x_ and (R_z_)_y_ values varied from 0.30 ± 0.05 µm to 0.78 ± 0.25 µm and from 0.31 ± 0.07 µm to 0.99 ± 0.12 µm, respectively.

### Migratory Behavior of Cells on Substrates With Graded Roughness

To study cell migration on roughness gradient substrates, the calculated migration velocities were compared in four different roughness ranges as shown in Figures 4I, 5I. For cells on G_40_ substrates, a gradual increase on migration velocity with higher roughness range was observed. The quantified velocity on the highest roughness ranges was 20.99 ± 6.5 μm/h, and the velocity was gradually reduced to 14.40 ± 5.54 μm/h, 9.63 ± 2.55 μm/h, and 5.88 ± 0.16 μm/h for the following 3 lower roughness ranges. The migration velocity on the highest roughness region was about 3.6 times faster than the cells on the lowest roughness region. In contrast, a different result was found for cells on the G_20_ substrate (Figure 5I). First, cells on higher roughness regions (0.75–0.85 µm) has the same range of velocity (18.56 ± 2.08 μm/h) with cells on G_40_ substrate in this roughness region. This migration velocity was 1.7 times higher than the cells on all three lower roughness ranges, which were 18.56 ± 2.08 μm/h, 11.99 ± 5.52 μm/h, 8.61 ± 2.93 μm/h, and 11.23 ± 2.72 μm/h from high to low roughness range. There was no significant difference among these three lower roughness regions. The average velocity of these three regions was 10.6 μm/h. In particular, the velocity in the 0.35–0.45 µm range was 1.9 times faster than the cells on the G_40_ substrate in the same range. This effect could attribute to the decreased stair width, and it suggests that cells sensed the adjacent roughness faster and took less time to migrate across the gradient. This result suggests that steepness of the graded roughness can be another topographic cue to modulate cell migration.

### Unidirectional Migration of MG 63 Cells Directed by Graded Roughness

Our experimental studies also showed that MG63 cells tend to migrate from higher to lower roughness region on both G_20_ and G_40_ substrates. Furthermore, cells tended to align along the direction of the roughness gradient during migration. To quantify this effect, the change in the orientation angle (dθ) was analyzed and is summarized in Figures 4J, 5J. It was found that cells tend to maintain their orientation once they align across the graded roughness and did not change their orientations. This can be observed from the high numbers of incidents for dθ = 0°. Furthermore, the range of dθ on graded substrates was much smaller than U_LR_ and U_HR_ shown in Figure 3G. The values of dθ were less than 24° for G_40_, whereas only a small number of incidents below 12° was observed for G_20_. This result clearly suggests that the cells tend to maintain their migration direction on a graded roughness with smaller step size.

Figures 4K, 5K show time-lapse images of MG63 cells that migrated on the G_40_ and G_20_ substrates, respectively. Their corresponding recordings are shown in the Supplementary Videos S3, S4 The image contrast was also relatively low due to light scattering induced from the ellipsoid voxels. We observed that cells on G_20_ were more aligned across the graded roughness than on G_40_ substrates. This trait can be observed from the traced frequency of the orientation angle of all the observed cells that migrated on these two substrates. Figures 4L, 5L summarized the analyzing results. It is noted that the range of angle of orientation (θ) was between −45° to 45°, which was much smaller than U_LR_ and U_HR_ (Figure 3F). Also, cells on G_40_ and G_20_ were almost perfectly aligned across the gradient for 42.56% and 62.8% of time for the entire migration period, respectively. This high dependency of cell orientation with respect to the roughness gradient can be observed from the traced trajectories of migrating cells, which are summarized in Figures 4M, 5M. It is noted that the 7 monitored cells migrated on G_20_ are separated into two figures in Figure 5M to better represent their orientations along the course of migration. 4 out of 7 cells aligned perfectly at 0° from the beginning to the end. Furthermore, some of these cells had a shorter total migration time as they were aligned well and had a higher migration velocity at lower roughness regions. It is also noted that cells on the G_40_ substrate changed their migration direction more often than the cells on G_20_, and their angle of orientation was between −45° to 45° as compared to G_20_ where the migration angle was nearly less than 15°. This observation could be attributed to the smaller stair width that allowed the cells to sense the steepness of roughness gradient and aided in better oriented migration. This effect supports the result that cells migrate faster on G_20_ than on G_40_ substrates.

Finally, Figures 4N, 5N show fluorescent micrographs of one of the migrating cells on G_40_ and G_20_ substrates, respectively. Surface roughness of the underlying graded substrate is labeled as ((R_z_)_x_, (R_z_)_y_). These figures clearly show that cells on both substrates have a highly polarized morphology that aligned across the graded roughness substrates. They have one or two narrowed lamellipodia that pointed toward the direction of the lower R_z_ regions. Also, cells have a long and slender tail that aligned with the graded roughness pattern with higher R_z_. Their F-actin structure shows high dependency on the roughness gradient where a leading edge is towards lower R_z_ and trailing end is from the higher R_z_. It is also noted that the cells on G_40_ spanned across three stairs, whereas the cells on G_20_ spanned across four to five stairs. These characteristic migrating morphologies clearly suggest that cells have a high unidirectional migratory behavior on roughness gradient substrates.

### Comparison of Migratory Behaviors of MG63 Cells on Uniform and Graded Substrates

**FIGURE 6:**
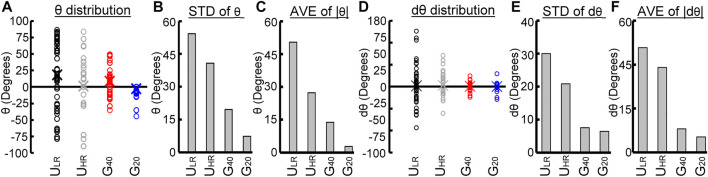
Comparison of cell migratory behaviors on the four substrates with different patterns of surface roughness. **(A)** Distributions of orientation angle (θ), **(B)** standard deviations of θ, **(C)** average of absolute θ, **(D)** distributions of change of orientation angle (dθ), **(E)** standard deviations of dθ, and **(F)** average of absolute dθ.

To illustrate the difference in the migratory behavior of cells on uniform (U_LR_ and U_HR_) and graded (G_40_ and G_20_) substrates, the distribution of the orientation angle θ and change of orientation angle dθ are compared. Figure 6A shows the distribution of orientation angle θ in open circles for each substrate, and the average of θ are represented by cross. It is clear from Figure 6A that the range of θ was large and random on U_LR_ and U_HR_ substrates. In contrast, the range was largely decreased for cells on G_40_ and G_20_ substrates, and G_20_ was much smaller than G_40_. To quantitatively compare their distributions, we studied the standard deviations of θ (STD of θ) and are shown in Figure 6B. It shows that the distribution of θ was much smaller on the graded substrate, and G_20_ had the narrowest range. The standard deviations of G_40_ and G_20_ were 19.6° and 7.2°, respectively. To further study the average migration orientation, we calculated absolute values of θ and compared their average values (AVE of |θ|). The results are shown in Figure 6C. It clearly shows that cells migrated on the G_20_ substrate had the lowest deviation from the graded direction. The average was only 2.84° when compared to 13.81° for cells on the G_40_ substrate.

To study the tendency of cells to change their migration direction, the distribution of change of the orientation angle dθ was also studied and is shown in Figure 6D. It shows that cells on the U_LR_ had the widest range of dθ, and cells on the U_HR_ had narrower range. This result suggests that cells on substrate with higher R_z_ had a higher tendency to maintain their migration direction. On the other hand, the range of dθ was much smaller for cells on G_40_ and G_20_ substrates. To quantify this effect, the standard deviation of dθ (STD of dθ) was also studied and is shown in Figure 6E. It clearly shows that cells on G_40_ and G_20_ had a much narrower range than the cells on U_LR_ and U_HR_, which were 7.6° and 6.5°, respectively. The average of absolute values of dθ (AVE of |dθ|) was also quantified. Figure 6F shows the analyzed results. It shows that the average values were much smaller for cells on the G_40_ and G_20_ substrates, which were 2.7° and 1.8°, respectively. It suggests that cells had a much lower deviation from the direction of graded substrate. Combining the results shown in Figures 6A–F, our experimental findings suggests that MG63 cells can sense the graded roughness substrate and have a high tendency to perform a unidirectional migration along the roughness gradient.

## Discussion

In this study, we evaluated a new topotactic cue, surface roughness (R_z_), on the migration of MG63 cells. Experimental findings on uniform substrates showed that MG63 cells have a slower migration velocity on a smoother surface (Figure 3E), and they also displayed a delayed onset of initial migration. The cell morphology on U_LR_ was more elliptical in shape with indistinct protrusions, whereas on U_HR_, the cell morphology was spindle-shaped with elongated filopodia, suggesting a higher migration tendency. These experimental results suggest that cells can determine their migrating velocity based on the roughness of their underlying substrates, and they do not have a preferred direction of migration on substrates with uniform roughness.

Our findings on uniform U_LR_ and U_HR_ substrates also implied that allowing a single cell to encounter different surface roughness on different parts of the cell body could potentially create a topographic cue to direct cell migration. To verify this hypothesis, we compared cell motility on G_40_ and G_20_ substrates. It was found that cells tend to move from high roughness to low roughness region, and migration velocity also decreased (Figures 4I, 5I). These results agreed well with our finding on U_LR_ and U_HR_ substrates and fortified the importance of surface roughness in influencing cell migration velocity. Interestingly, different from the G_40_ substrate, where migration velocity gradually decreased when moved to lower roughness region (Figure 4I), the cells on the G_20_ substrate showed no significant difference in velocity on the three lower-roughness regions (Figure 5I). In addition, cells on the lowest roughness range of G_20_ substrates also migrated 1.9 times faster than cells on G_40_ substrates. Hence, G_20_ that has shorter stair size, can provide a stronger topotactic cue than G_40_ substrate for directing MG63 cell migration. This finding suggests that topotactic substrate with a steeper increment of roughness could promote faster cell migration.

In summary, our experimental findings suggest that there are three unique migratory behaviors of MG63 cells on roughened substrates. First, MG63 cells migrate faster on a substrate with a higher R_z_ value. Second, MG63 cells prefer to migrate from higher to lower roughness regions. Third, migration velocity can be high on a graded substrate with a steeper roughness gradient in low R_z_ range. These results suggest that surface roughness could be another topographic cue for topotaxis.

To highlight the difference between this roughness gradient-dependent cell migration and previously reported studies on topotaxis, we compare our work with representative topotactic papers in Table 1. Kim et al. (2009a) and Park et al. (2016) are the two pioneer works that used micro- or nano-structures to create topographic patterns with different spatial densities to demonstrate cells can have a unidirectional migration. The primary design criterium was to use a fixed dimension of a surface feature made of UV-curable polyurethane acrylate (PUA) and varied the inter-feature distance to create a topographic gradient. Kim et al. (2009b) showed that NIH3T3 cells tend to migrate from sparse to dense areas, whereas Park et al. (2016) showed that melanoma cells tend to migrate from denser to sparser areas. Using a 5 µm or 10 µm wide strips of PDMS nanopillars or nanoholes with intermediate flat surfaces, Cheng et al. (2021) showed that MC3T3-C1 cells have a bidirectional migration along the strips with nanostructures. These studies suggest that cells can sense surface topographic features and determine their migration direction. In contrast, Sochol et al. (2011) studied the migratory behaviors of bovine aortic endothelial cells (BAECs) on a gradient of PDMS microposts with different aspect ratios and a fixed interpost spacing, which were used to simulate different levels of stiffness. Using microposts with different diameters to create a substrate with a gradient of stiffness, they found that BAECs tend to migrate from lower to higher stiffness region.

**TABLE 1:** Comparison of topographic patterns for topotaxis.

	Material	Topotactic feature	Spatial variation	Cell type	Migration direction
Kim et al. (2009b)	Polyurethane acrylate (PUA)	Ridges	Inter-ridge intervals: 4.5–9.5 μm	NIH3T3	Unidirectional sparse-to-dense areas
		W = 1.5 μm, 2 μm			
		H = 500 nm			
Park et al. (2016)	Polyurethane acrylate (PUA)	Nanoposts	x: 0.3–4.2 μm	Melanoma cells	Unidirectional dense-to-sparse areas
		D = 600 nm	y: 600 nm		
Sochol et al. (2011)	Polydimethylsiloxane (PDMS)	Microposts	Interpost spacing: 2 μm	Bovine aortic endothelial cells	Unidirectional lower-to-higher stiffness
		D = 2–6 μm			
		H = 7 μm			
Cheng et al. (2021)	Polydimethylsiloxane (PDMS)	Nanopillars: D&H = 250 nm, 500 nm, 700 nm	Uniform nanostructure grating: 5 μm or 10 μm wide	MC3T3-E1	Bidirectional along gradient patterns
		Nanoholes: D&H = 250 nm, 500 nm, 700 nm	Pitch between gratings: 5 μm or 10 μm		
This study	Polypropylene (PP)	Surface roughness: R_z_ = 0.29–1.11 μm	Voxel distance (VD)	MG63	Unidirectional high-to=low R_z_
			x: 0.571–0.836 μm		
			y: 0.571–0.836 μm		

In 2018, Park *et al.* pointed out that the direction of topotactic migration could attribute to the differential penetration of cells into areas between surface structures. Softer cells can penetrate more into space between surface features in sparser regions and have a higher level of cell–ECM interaction. This concept suggests that stiffer cells tend to migrate toward denser-fiber zone while soft cells tend to migrate toward sparser-fiber zone. Considering the extracellular matrix is a 3D structure that do not have flat areas, we used the TPP method to create a more biomimetic substrate that can provide a uniform micron to submicron features without flat area. This substrate can eliminate the complications from the inter-feature flat area that have the flat-to-feature ratio varied from two to seven times (Kim et al., 2009b; Park et al., 2016), where flat areas contribute larger portion in sparser regions. Furthermore, the created 3D topographic substrate is uniform in both *x*- and *y*-direction. It can eliminate spatial non-homogeneity of having to increase inter-feature distance in the *x*-direction while having to maintain the same distance in the *y*-direction to study unidirectional cell migration (Park et al., 2016). Last, the influence of deformability of surface features can be eliminated. This was achieved by using a high stiffness nanoimprinted PP substrate (1.32 GPa) and the ellipsoid-shape voxels. This design can effectively preclude the complication contributed by the deformability of an elastic surface features made by softer materials, such as PUA and PDMS, which are in a range 0.5 MPa to several MPa.

As MG63 cells have lower stiffness due to the reduced organization of their cytoskeleton (Wang et al., 2016), they could penetrate differentially into the spaces of topographic features. However, different from Park et al., 2016, our substrates are isotropic, with equal density of roughness. Thus, the space for penetration was in the same range on all roughness ranges. However, the penetration distance varied with R_z_. On lower R_z_, MG63 cells could maximize their cell–ECM interactions since the depth from peak to valley is small. Moreover, our experimental findings show that MG63 cells tend to migrate from higher to lower R_z_ regions, without reversing direction during migration period. This result suggests that high R_z_ could be directing cells differently, even though penetration space is close to lower R_z_. In addition, the higher migration velocity on substrate with high roughness gradient in lower roughness region is another interesting finding. Hence, our observations are unique and suggest that the mechanisms of cell migration on roughened substrates could be completely different from the previously reported substrates. Also, it will be interesting to investigate migration behavior of cells with different stiffness on roughness gradients to elucidate the mechanism of roughness-induced topotaxis. Further studies are needed to explore the effect of roughness on cell migration.

## Conclusion

The advanced 3D lithography method, two-photon polymerization, offered a high precision technique to create substrates with programmable topography down to micron and submicron scale. With the aid of this technology, we expanded our study on topotaxis with precisely defined roughness. Combining the nanoimprint process, TPP-made topographic patterns can massively replicated onto PP films for larger-scale quantitative studies. Although there were some limitations to both TPP and nanoimprint processes, we still successfully fabricated four different types of substrates to investigate the contact guidance of topographical roughness on MG63 cell migration. It was verified that different surface roughness ranging from 0.29 ± 0.2 μm to 1.11 ± 0.26 μm can be created by controlling voxel distance. Currently, the total area that can be fabricated by our TPP setup was 400 μm by 200 μm. It can monitor only one to two cells per experiment, which results in a lower number of cells can be investigated in this study. This deficiency can be increased by using a precision motor with a wider operation range, or using a stamping machine to nanoimprint multiple roughness patents on one PP substrate.

This study is also the first study that designed an isotropic and continuous topographic feature of gradually varying surface roughness (R_z_) to promote unidirectional cell migration. We identified two major parameters of a R_z_ gradient in influencing cell migration: first is the level of R_z_ and second is the steepness of adjacent roughness, i.e., stair sizes. A higher steepness promoted faster and more directed migration of the MG63 cells. This work can be explored further to understand the role of roughness in biasing cell migrations. The methods presented in this study for substrate fabrication, *i.e.*, two-photon polymerization and nanoimprint, could become powerful tools to design biomimetic topographical substrates that can mimic the effect of environmental stimuli or signaling in a 3D cell environment and advance our understanding of cell–ECM interactions.

## Data Availability

The raw data supporting the conclusion of this article will be made available by the authors, without undue reservation.
