# Supplementary material for: Programmed Topographic Substrates for Studying Roughness Gradient-Dependent Cell Migration Using Two-Photon Polymerization
Source: Front Cell Dev Biol. 2022 Mar 22;10:825791. doi: 10.3389/fcell.2022.825791 (PMC8980465; doi:10.3389/fcell.2022.825791)
Supplement: Supplementary file 2 [file DataSheet1.PDF]

Programmed Topographic Substrates for Studying Roughness-Gradient-Dependent Cell Migration Using Two Photon Polymerization

Subhashree Shivani,[1+] Yu-Hsiang Hsu,[1+†]* Cheng-Je Lee,[1] Chi-Sheng Cheong,[2] Tien-Tung Chung,[2†] An-Bang Wang, [1†*]

[1] Institute of Applied Mechanics, National Taiwan University No. 1, Sec. 4, Roosevelt Rd., Taipei, 106, Taiwan (R.O.C)
[2] Department of Mechanical Engineering, National Taiwan University No. 1, Sec. 4, Roosevelt Rd., Taipei, 106, Taiwan (R.O.C)
+These two authors contributed equally to this work.
[†]These three authors contributed equally as senior authors to this work.
***Correspondence:**
Yu-Hsiang Hsu yhhsu@iam.ntu.edu.tw (Topotaxis);
An-Bang Wang abwang@iam.ntu.edu.tw (TPP)

1 Supplementary Figures

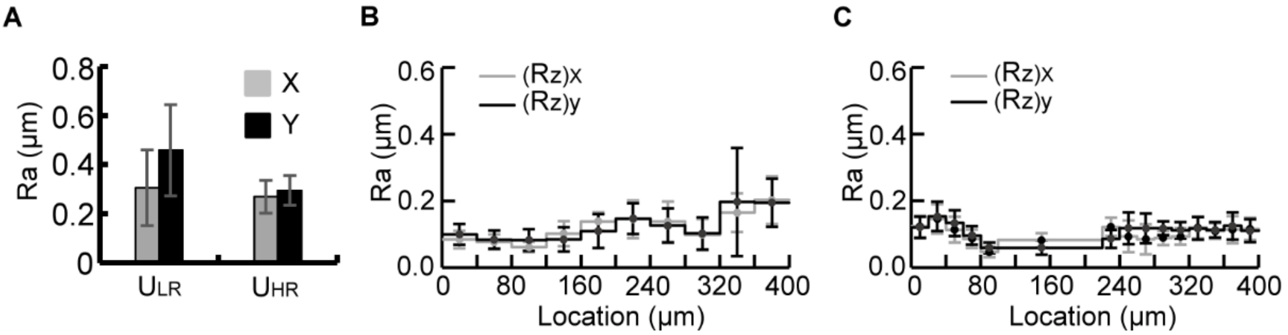

Supplementary Figure S1. Measured R_a values of PP substrates for U_{LR} and U_{HR} (A), G_{40} (B), and G_{20} (C).
